# Application of Fibre Bragg Grating Sensors in Strain Monitoring and Fracture Recovery of Human Femur Bone

**DOI:** 10.3390/bioengineering7030098

**Published:** 2020-08-19

**Authors:** Ali Najafzadeh, Dinusha Serandi Gunawardena, Zhengyong Liu, Ton Tran, Hwa-Yaw Tam, Jing Fu, Bernard K. Chen

**Affiliations:** 1 Department of Mechanical and Aerospace Engineering, Monash University, Melbourne, VIC 3800, Australia; Jing.fu@monash.edu (J.F.); Bernard.chen@monash.edu (B.K.C.); 2 Photonics Research Center, Department of Electrical Engineering, The Hong Kong Polytechnic University, Hong Kong, China; Zhengyong.liu@connect.polyu.hk (Z.L.); hwa-yaw.tam@polyu.edu.hk (H.-Y.T.); 3 Department of Orthopaedic Surgery, Monash University, Melbourne 3000, Australia; Ton.Tran@monashhealth.org

**Keywords:** Fibre Bragg Grating (FBG) strain sensor, thighbone (femur), fracture recovery, biomechanics, in vitro bone testing

## Abstract

Fibre Bragg Grating (FBG) sensors are gaining popularity in biomedical engineering. However, specific standards for in vivo testing for their use are absolutely limited. In this study, in vitro experimental tests were performed to investigate the behaviors and applications of gratings attached to intact and fractured thighbone for a range of compression loading (<300 N) based around some usual daily activities. The wavelength shifts and the corresponding strain sensitivities of the FBG sensors were measured to determine their effectiveness in monitoring the femoral fracture healing process. Four different arrangements of FBG sensors were selected to measure strains at different critical locations on the femoral sawbones surface. Data obtained for intact and plated sawbones were compared using both embedded longitudinal and coiled FBG arrays. Strains were measured close to the fracture, posterior linea aspera and popliteal surface areas, as well as at the proximal and distal ends of the synthetic femur; their responses are discussed herein. The gratings on the longitudinally secured FBG arrays were found to provide high levels of sensitivity and precise measurements, even for relatively small loads (<100 N). Nevertheless, embedding angled FBG sensors is essential to measure the strain generated by applied torque on the femur bone. The maximum recorded strain of the plated femur was 503.97 µε for longitudinal and −274.97 µε for coiled FBG arrays, respectively. These project results are important to configure effective arrangements and orientations of FBG sensors with respect to fracture position and fixation implant for future in vivo experiments.

## 1. Introduction

Femoral fracture is a life-threatening injury, usually caused by high energy impact and associated with major local soft-tissue injury, as well as other more serious axial damage [1,2,3,4,5,6,7]. Early surgical stabilization is now the standard treatment for this injury to allow early mobilization and rehabilitation [8,9,10,11,12,13,14]. The usual construct is either an intramedullary device or a plate to achieve stability at the fracture zone [1,12,15,16,17,18,19,20,21]. 

Recovery of a fractured tibia can take up to several weeks or a few months. It is difficult to ascertain the extent of fracture recovery and the optimum time for the patient to safely resume normal load-bearing activities. Although CT scans can provide a rough measure of fracture recovery, they expose the patient to harmful radiation. In this paper, the use of Fibre Bragg Grating (FBG) sensors to monitor bone strain under applied load is investigated as a potential method to assess the strength of the bone at various stages after surgery in order to determine an optimal point at which patients can resume load-bearing activities.

Various techniques, gauges and sensor types are utilized to measure strain and stiffness in bone and orthopaedic implants [22,23]. These sensors include conventional resistive strain gauges, as well as those that employ optical fibres with Fibre Bragg Gratings (FBGs). In vitro studies were performed on implants in order to understand their role on bone fracture fixation, and also to evaluate their designs for in vivo investigations to monitor strains under various loading conditions [24]. However, application of strain analysis sensors throughout the bone fracture recovery assessment process is uncommon and mostly limited to conventional resistive strain gauges. 

The use of FBG sensors in biomechanics applications for bone–prosthesis interface strain measurements is advantageous, as conventional resistive strain gauges may cause damage due to electrical circuity [25]. Although conventional resistive sensors can measure the average strain between two specific points or insertion sites within a ligament, FBG sensors provide strain over the entire tissue by using multiplexing techniques [26]. An observed Bragg wavelength shift makes this induced strain monitoring possible, as the wavelength is proportional to the grating period. The temperature and strain sensitivities of an FBG sensor recorded at 1550 nm are approximately 13.7 pm/°C and 1.2 pm/µɛ, respectively [27]. FBG sensors also have an added benefit due to their low conductivity, which makes them ideal for clinical applications. The multicore optical sensors are also utilised in shape-sensing of medical instruments, including catheters, by calculating the twists in fibres based on the measured longitudinal strains in each fibre core and performing 3D shape reconstruction of the objects [28,29,30,31].

Previous work was reported by Talaia [32], in which seven FBG sensors were secured on the surface of a stainless steel bone implant and attached to a synthetic femur for fracture fixation purposes. Mishra et al [33] demonstrated the use of FBG sensors in the categorization of the decalcification stages of bone. The in vitro response of bone strain under particular site loading indicated the bone stiffness as well as the degree of osteoporosis. Micromotion (microstrain) assessments during in vitro experiments are helpful to understand implant design performance during in vivo trials [34,35,36,37,38]. Load-transfer studies within a resurfaced femur were previously used to investigate the effects of mechanical factors on potential mechanisms of failure, including stress shielding and early femoral fractures [39,40,41,42,43,44,45]. Sideways load configurations were also considered and analysed to measure the in vitro strain distribution in the proximal human femur [46,47,48]. Any micromotion within the range of 100–150 µm is considered to be a sign of aseptic loosening of an implant. Cristofolini et al. [49] studied the development of in vitro protocols in order to measure micromotions of prostheses–bone in resurfaced femur. Shear slippage and gap opening micromotions were measured at two different locations, the first on the posterior side between the prosthesis and the bone and the second between the most medial points of the prosthesis rim. 

Future research is required to classify the global bone response and increase in vivo applications of this FBG sensor technology. Here, the applications of FBG sensors are investigated with focus on the sensitivity and simplicity of installment and the response, interrogation and implementation of in vitro bone-implant strain analysis on composite synthetic fractured femur. This study investigates effective arrangements for the FBG sensors arrays and examines practical sensors orientations for future in vivo applications.

## 2. Methods

### 2.1. Theory of FBG Sensors

The core and cladding of an optical fibre have different refraction indices. Stretching an FBG alters its grating period, which in turn leads to a change in the Bragg wavelength. This alteration is related to variation of the refractive index modulation spatial period using Bragg’s law [50]:

(1) $$\lambda_{B}=2n_{eff}\Lambda$$

where $\lambda_{B}$ represents the Bragg wavelength, Λ is the period of the grating and $n_{eff}$ is the effective refractive index of the fundamental mode propagating in the fibre core. The Bragg wavelength shift depends on the temperature change (Δ*T*) and the longitudinal strain (ε) according to Equation (2).

(2) $$\Delta \lambda_{B}=K_{T}\cdot \Delta T+K_{\varepsilon }\cdot \varepsilon$$

where $\Delta \lambda_{B}$ is the change in Bragg wavelength, Δ*T* is the temperature alteration, ε is the local strain and *K_T_* and *K_ε_* are constants [51]. When thermal variations and/or mechanical perturbations take place, the reflected Bragg wavelength alters according to Equation (3).

(3) $$\Delta \lambda_{B}=2\left(\Lambda \frac{\partial n_{eff}}{\partial T}+n_{eff}\frac{\partial \Lambda }{\partial T}\right)\Delta T+2\left(\Lambda \frac{\partial n_{eff}}{\partial \varepsilon }+n_{eff}\frac{\partial \Lambda }{\partial \varepsilon }\right)\varepsilon_{z}$$

The thermal effect and the strain dependence on the Bragg wavelength are shown by the first and second terms, respectively; therefore, an FBG can be employed as a temperature as well as a mechanical strain sensor.

A deformation in the longitudinal direction can affect both Λ and $n_{eff}$, the former by altering the pitch of the grating and the latter by the effect of photoelasticity. This results in a variation in $\lambda_{B}$, which is related to the applied longitudinal strain.

(4) $$\frac{\Delta \lambda_{B}}{\lambda_{B}}=\left(1-\rho_{e}\right)\varepsilon_{z}$$

where $\rho_{e}$ is the elasto-optic coefficient from which the strain can be calculated. Equation (3) can be expanded as follows, where ρ_ij_ is Pockel’s coefficient of the stress-optic tensor and υ is Poisson’s ratio [27,52]. For this research project, the values used for these coefficients are p_11_ = 0.113 and p_12_ = 0.252, and the Poisson’s ratio is considered to be υ=0.16 [27].

(5) $$\frac{\Delta \lambda_{B}}{\lambda_{B}}=\left(1-\frac{n^{2}{}_{eff}}{2}\left[\rho_{12}-\upsilon \left(\rho_{11}+\rho_{12}\right)\right]\right)\varepsilon_{z}$$

Cadaver and composite femurs were compared with each other regarding their biomechanical properties [53,54], with the overall recommendation that fourth-generation composite femur (4GCF) sawbones should be utilised when representing the biomechanical properties of femurs in youth. In this study, biomechanical tests were conducted on a 4GCF sawbone, as this offered a stable bone–prosthesis construct with increased fatigue and fracture resistance to test the sensitivity level of FBG sensors in the femur bone surface strain measurement. A standard silica single mode synthetic mineral optical fibre (SMF) with 125 µm and 8 µm cladding and core diameters, respectively, was used to measure the strain on the sawbones surface. The coating material of the optical fiber was acrylate and the diameter of the optical fiber, together with its buffer for the nonsensing region, was ~250 µm. Four strands of optical fibres, each consisting of an FBG array, were used to carry out the experiments. Two FBG arrays, each consisting of three and five FBGs, respectively, were used for the intact femur, where the array consisting of three FBGs was placed longitudinally along the femur and the array with five FBGs was coiled around the bone. The other two FBG arrays, with five and seven FBGs, were attached in longitudinal and coiled orientations on the plated sawbones sample. A 248 nm excimer laser together with phasemasks (Ibsen Photonics, Farum, Danmark), with the pitch ranging from 1055 nm to 1091 nm, were utilised during FBG inscription. Gratings 10 mm in length were fabricated using the beam scanning technique. 

### 2.2. Instrumentation, Testing and FBG Fixation

**Figure 1:**
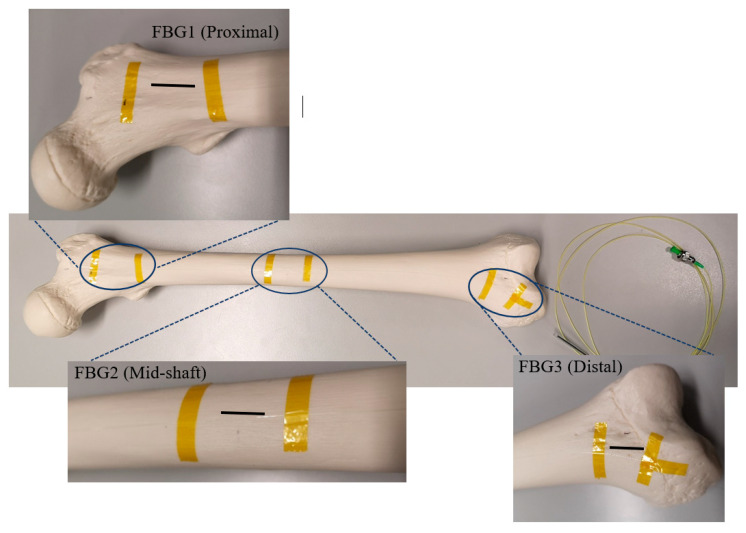
Intact femur with the secured Fibre Bragg Grating (FBG) array, including three FBGs.

The arrangement of each FBG on the FBG array was based on monitoring of the strain distribution through the bone surface, in particular around the midshaft and fractured area. Figure 1 shows the first arrangement in which the intact femur was tested with the attached FBG array, including three gratings near the proximal, middle shaft and distal areas of the sawbone. The sensors were anchored on the bone surface using Loctite 431 adhesive glue. Kapton tape was used as extra fixation and also to mark the locations of the sensors. Two FBG sensors were attached on the proximal and distal ends of the femur at 2 cm and 38 cm from the hip joint, respectively. The middle shaft sensor was located 20 cm from either end of the femur. In the second test, an FBG array was coiled around the intact femur bone. The first grating was placed 2 cm from the hip joint and the other four gratings were attached on the femur at an angle of 30°, with distances of 18 cm, 20 cm, 22 cm and 32 cm, respectively, from the hip joint. 

**Figure 2:**
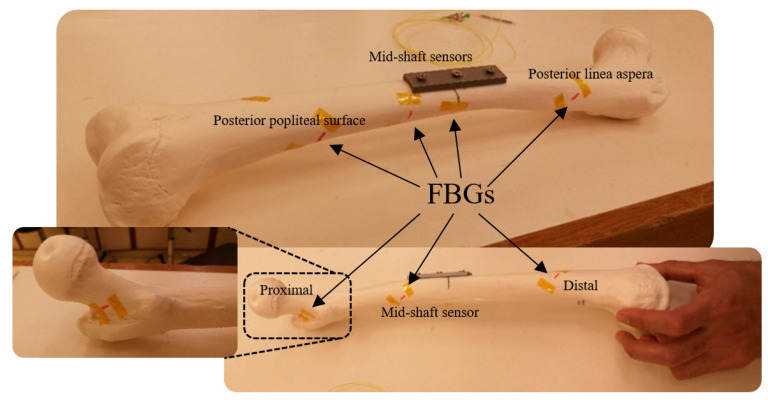
Plated femur sawbones instrumented with seven FBG strain sensors on a coiled FBG array.

In the next experiment, a linear greenstick fracture with an angle of 30° (with respect to horizontal line) was introduced to the midshaft of the femur sample using a surgery saw. Then, the fracture was fixed with a three-hole implant plate. During loading of the fractured bone, a third optical fibre was anchored on the implant side with five FBG strain sensors on the grating array. Three gratings were attached close to the fracture in the middle shaft to measure strain distribution in that particular area. The FBG sensors were positioned longitudinally at 18 cm, 20 cm and 22 cm, with respect to the hip joint. The other two FBG sensors were placed at the two ends of the sawbones to compare the behavior of the femur with that of the intact one. In the final test, a coiled FBG array with seven gratings was employed (Figure 2). The positions of the sensors with respect to the proximal end of the femur were 2 cm, 9 cm, 15 cm, 20 cm, 23 cm, 29 cm and 32 cm, which were attached at the same angle as that in the second test (Table 1).

**Figure 3:**
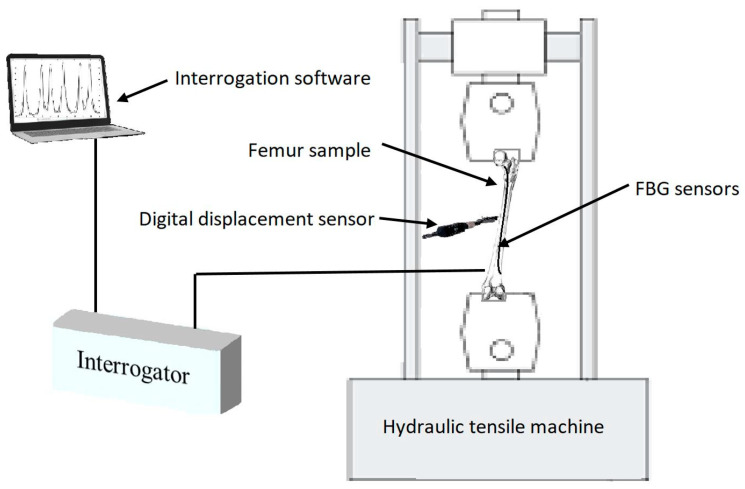
Schematic view of the experimental setup.

Compression loading was introduced to the femur sawbones model to measure the shift of Bragg wavelengths of the attached FBGs. The magnitude of loading was maintained at a relatively low level to prevent any damage or failure on the sample [55,56,57,58]. Given the sensitivity level of FBG sensors in comparison to conventional strain gauges, the wavelength shift of these sensors could still be detected under a small loading amount. The strain sensitivity of an FBG was 1.1 pm/µɛ. The reflection spectra of these FBGs were monitored using an interrogator (Micron Optics: sm125) using the inbuilt program containing the peak detection algorithm [59,60] with a resolution of 1 pm, and recorded using a LabVIEW program throughout the experiment. The same sawbones was loaded during four test stages considering the strategy of single specimen [57,58,61,62,63]. During each stage, midshaft horizontal displacement of the composite femur was controlled and monitored along with the load applying process. A Mini Instron hydraulic tensile machine with a loading capacity of 2 kN was used, and the vertical displacement rate was controlled during the tests at 1 mm/min. A schematic view of the experimental setup is illustrated in Figure 3.

The maximum loading times for the intact and plated femur were 57.30 s and 58.60 s, respectively. Compression loading values were recorded for each step at every millimeter of femur midshaft point displacement. Maximum loading during the entire test did not exceed 300 N (Table 2).

## 3. Results 

**Figure 4:**
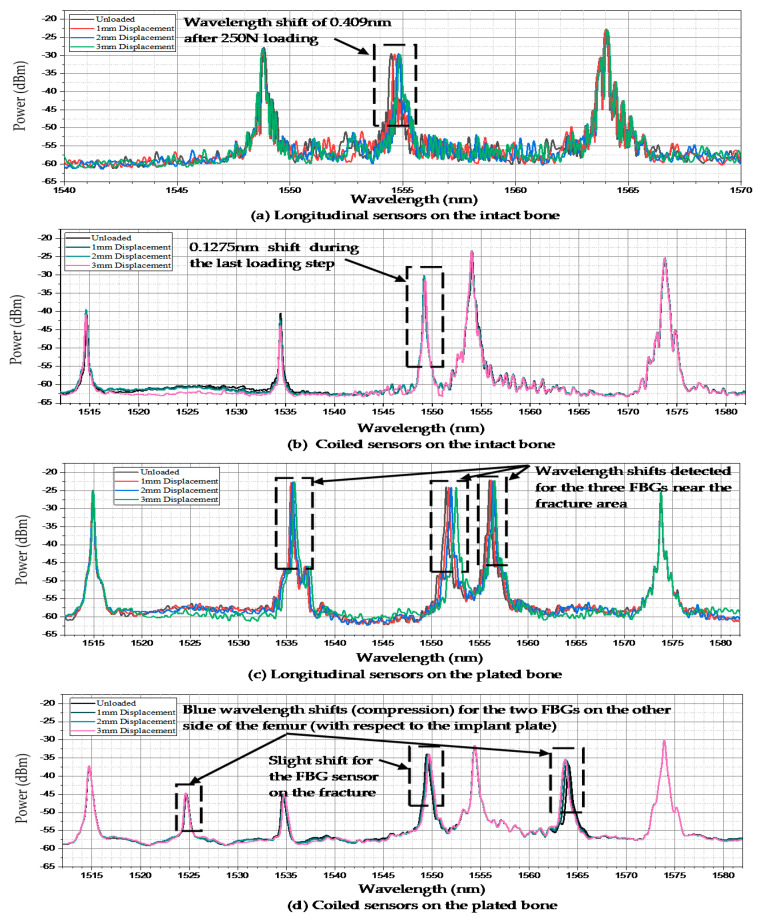
Wavelength shifts of the FBG arrays anchored on the femur sawbones. (**a**) Longitudinal sensors on the intact bone; (**b**) Coiled sensors on the intact bone; (**c**) Longitudinal sensors on the plated bone; (**d**) Coiled sensors on the plated bone.

The wavelength spectrum after the first loading test process demonstrated that the highest wavelength shift occurred in the middle shaft sensor. The Bragg wavelength shifted from 1554.495 nm to 1554.904 nm after three steps of compressed loading. However, this shift was not noticeable in the other two FBGs, as shown in Figure 4a. The second test on the coiled FBG array of intact femur represented that the response of sensors to loading was relatively minor regarding the orientation of fixed gratings. Figure 4b shows that only the middle strain sensor reaction was detectable during the last loading step, which shifted by 0.1275 nm. During the third test, which was implemented on the plated sawbones, three FBG sensors near the fractured area presented dramatic wavelength shifts. According to Figure 4c, the peak wavelengths of the second and fourth gratings shifted by 0.341 nm and 0.471 nm, respectively. The maximum shift of wavelength of the third FBG was recorded by the middle shaft sensor, which was 0.544 nm. In the last test, seven peaks were monitored for the sensors on the coiled FBG array on the plated femur. The wavelength shift of the fourth FBG was 0.24 nm. Two FBG sensors recorded negative strain (compression) where their wavelengths showed blue shift, as shown in Figure 4d. The wavelength shifts noted for these two sensors were −0.07 nm (posterior linea aspera FBG) and −0.415 nm (posterior popliteal surface FBG).

**Figure 5:**
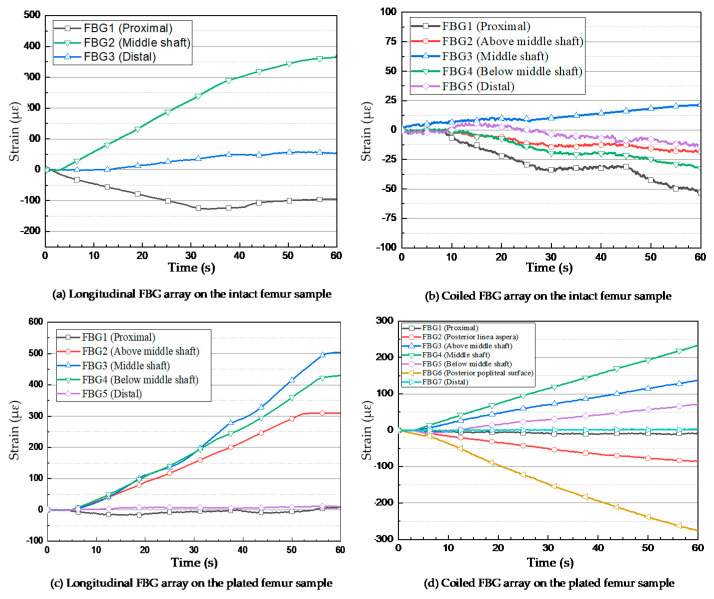
Strain measurements of FBG sensors under the applied compression loads. (**a**) Longitudinal FBG array on the intact femur sample; (**b**) Coiled FBG array on the intact femur sample; (**c**) Longitudinal FBG array on the plated femur sample; (**d**) Coiled FBG array on the plated femur sample.

During the entire test process, all FBG sensors were firmly anchored to the bone surface and the wavelengths shifts were recorded. The behaviors of all sensors were observed during each test and their wavelength shifts were compared to each other. One FBG array was used during each compression loading test to simulate the conditions of future in vivo tests. Figure 5a shows the response of the FBGs to different loading steps during the first experiment. A maximum strain of 372.2 µɛ was measured by the middle shaft sensor. During the first and second loading steps, which consumed a time duration of about 24.88 s and 41.38 s, respectively (Table 2), the strain level increased to 144.34 µɛ and 265.95 µɛ. The strain values of the other two sensors at the distal and proximal areas were considerably lower than that of the femur middle shaft.

For the intact femur sawbones, the strain values were measured by the FBG sensors on the coiled FBG array. The embedded optical fibre contained five gratings, among which the nearest FBG to the femur centre point measured a strain level of 21.47 µɛ, as shown in Figure 5b. The nearest FBG sensor to the femur proximal area recorded −53.3 µε. The other three sensors detected relatively lower and negative (compression) strain values. 

**Figure 6:**
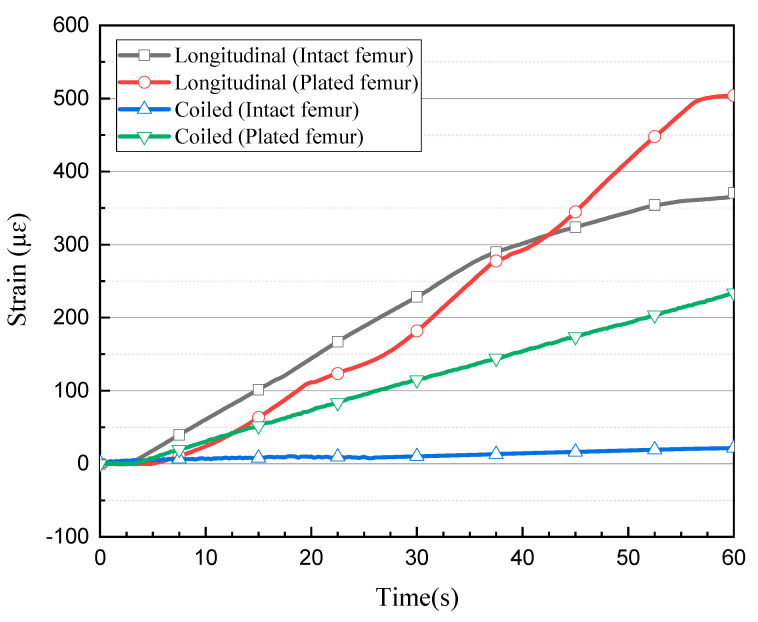
Comparison of strain level measured by the central FBG sensor during each test.

The first two loading tests were repeated after introducing an angled fracture to the femur sample. The new longitudinal and coiled FBG arrays, consisting of five and seven FBGs, respectively, were incorporated to measure the strain values more accurately on and around the implant area. In the longitudinal fibre, three sensors were close to the fracture; their respective strain measurements are shown in Figure 5c. The FBG sensor on the fracture line demonstrated a higher amount of strain rather than the other two sensors placed with a distance of 2 mm on each side. During the first and second loading steps, the strain of the middle shaft sensor increased to 124.44 µɛ and 287.81 µɛ, while this amount did not go beyond 108.78 µɛ and 87.25 µɛ during the first loading step and 244.81 µɛ and 181.82 µɛ during the second loading step, as observed from the fourth and second sensors, respectively. The maximum strain value recorded by the third (middle) sensor exceeded 503.97 µɛ, which was much higher than the value measured by middle shaft sensor on the intact femur (Figure 6). Similar to the first loading test, the two FBGs anchored on the two ends of the bone measured much lower strain during the loading steps.

The orientation and location of each sensor demonstrated considerable effects on the strain recorded in a coiled FBG array. Figure 5d shows that the central grating measured the strain of 234.11 µɛ during the fourth loading test. The maximum level of strain according to the orientation of FBG sensors was recorded by the sensor anchored at the posterior popliteal surface, which was −274.97 µε. Similar to the previous tests, this strain measurement showed lower amounts of strain for the other FBGs. Two FBG sensors located on the other side of the bone (with respect to the implant plate) at the posterior linea aspera and the popliteal surface areas of the composite femur, recorded negative strain (compression) based on the type of force the FBG experienced at those positions. As expected, the minimum measured strain belonged to the two sensors at the two ends of the femur bone, which did not exceed 3.7 µɛ and –9.9 µɛ for the proximal and distal ends, respectively.

## 4. Discussion

In this study, various arrangements and orientations of FBG gratings on a single optical fibre were evaluated to assess their applicability of bone surface strain measurement to monitor healing of a fractured femur. This was achieved by comparing cortical bone strain levels after applying compression loads on both intact and implanted femur. Four steps of in vitro experimental studies were performed using a single fourth-generation composite femur 4GCF sawbones, during which the thighbone was instrumented with FBG arrays involving different numbers of gratings attached at specific critical bone surface locations at different angles. The primary aim was to assess the feasibility of cortical bone strain measurement as a means of assessing the extent of recovery of the patient’s fractured bone after orthopaedic implantation surgery. 

Any change in strain level as a result of bone displacement under a given load is related to the density of the recovering bone and its relative strength to a neighboring implant area [64,65]. Premature resumption of load-bearing activities may be harmful and could adversely affect the bone-healing process. As a result, at different stages of the recovery process, any type of load-bearing activity should be controlled and the consequent strain level should be monitored to prevent overloading or damage, which could delay the recovery process. Suitable load-bearing activities could be gradually prescribed to optimise the time of fracture recovery.

The biomechanical application of FBG sensors, particularly in fracture healing assessment procedures, is demonstrated by the derived data. Given their sophisticated level of sensitivity in comparison with conventional resistive strain gauges, they may be reliable substitutes in strain measurement during the bone and tissue recovery process. While conventional strain gauges measure strain only at specific points, an embedded FBG array with a sufficient number of sensors is capable of providing strain values throughout the bone surface. Furthermore, they are relatively lighter, smaller and easier to embed, as well as being more sensitive and resistive to electromagnetic fields. FBG sensors in silica optical fibers can remain in the human body during in vivo experiments and can also be inscribed in biocompatible polymer optical fibers, whereas extended use of conventional resistive strain gauges in the body increases the risk of toxicity and infection. This study provides useful insight into the strain response of FBG sensors in the proximity of a fracture (crack) and implant plate, as well as their orientation and angle with respect to the crack and the location and amount of loading on the femur sawbones. 

To date, no standardised method exists to assess the fractured femur bone recovery process. The results obtained in this in vitro experimental research could be used in future experiments to study strain distribution at selected positions on the cortical bone of a fractured femur after orthopaedic surgery, thereby providing a method of determining the extent of bone recovery so that the patient may return to load-bearing activities as soon as it is safe. These future trials could involve the use of FBG sensors oriented longitudinally and angularly surrounding the fractured area under various loading conditions (axial load, bending and torsion) on the human femur to study the strain response of the gait cycle, daily work or professional athletic activities. The data could provide an appropriate and safe timeframe to initiate various activities and may help to specify the maximum amount of load each patient can apply to the injured area, as well as relevant safe movements after each follow-up visit with the surgeon. This is expected to dramatically shorten the recovery period for specific cases and prevent fatigue damage, overloading and subsequently bone nonunion or fracture recurrence after initiation of activities by the patient. 

Further to fractured femur bone recovery assessment application, FBGs can also be used for long-term bone microstrain and displacement measurement. Due to the strain shielding effect in femur bone under different activities and applied loads after orthopaedic surgery [65], as well as uncertainties in material properties and loads in femur computational models [66], understanding strain distribution on the surface of a bone over long periods of time is of high interest. When orthopaedic implants are left in the body after the recovery process in order to prevent a second surgery, there is also a risk of bone loss due to stress shielding [65,67,68]. Bone loss of 3–21% was reported in fractured femur and tibia bones in comparison to nonfractured ones [69,70,71]. In osteopenic patients, this loss represents a huge risk for fracture, specifically for that of the femoral neck [72]. On the other hand, implant removal after completion of the healing period is associated with the usual risks of surgical treatment. Therefore, the pros and cons of implant removal surgery should be balanced to subsequently optimise the timing of this second surgery to prevent fracture recurring in the same area. This balance and timing can be obtained through accurate and reliable strain measurement using FBG sensors.

When only strain values are required during in vivo experiments, temperature cross-sensitivity of the sensor must be considered under controlled conditions, particularly for in vivo applications under vigorous physical activity or for infectious processes, which are followed by a rise in temperature and can dramatically influence the recovery outcome. A multicore optical fibre can be secured on the bone during future in vivo trials. If the axial strain is negligible during compression loading, then the central core’s wavelength shift can be measured and considered for temperature change [28,31].

## 5. Conclusions

Previous studies employed fibre optic sensors to replace conventional strain gauges in biomechanical applications, including intervertebral disc bulging and cadaveric mandible strain measurements [73,74]. However, very limited studies exist regarding clinical or biomechanical experiments assessing human femur middle shaft fracture recovery, especially by the use of single fibre optic strain sensors on the cortical bone. Here, various sensor arrangements and orientations of FBGs on critical sites were studied, including the anchoring angle during the midshaft implanted femur bone healing procedure. However, the strain response may vary depending on different angles, shapes and sizes of fractures, including oblique, impacted and spiral, and also according to their locations on the thighbone surface. As a result, strain levels should be measured on various femur bone fracture types and obtained data should be compared. Other limitations, including access to the surface of the bone at required areas during orthopaedic surgery, also require focus. 

Further in vitro investigations are required to test whether FBG arrays with a combination of both longitudinal and lateral gratings are desirable during the healing process and to investigate their responses on femur bones with different types of middle shaft fractures, including oblique, impacted and spiral.

## Figures and Tables

**Table 1:** Distance of FBG sensors with respect to the proximal end of the composite femur on each FBG array.

	Distance of Gratings on the Optical Fibre (cm)			
	Intact		Plated	
FBG Sensors	Longitudinal Fibre	Coiled Fibre	Longitudinal Fibre	Coiled Fibre
FBG1	2	2	2	2
FBG2	20	18	18	9
FBG3	38	20	20	15
FBG4	-	22	22	20
FBG5	-	32	38	23
FBG6	-	-	-	29
FBG7	-	-	-	32

**Table 2:** Compression loading test parameters on femur sample during four different steps.

Femur Sawbones	FBG Array	Midshaft Displacement (mm)	Total Vertical Displacement (mm)	Loading Amount (kN)	Loading Time (s)
Intact Femur	Longitudinal Fibre	1.01	0.414	0.098	24.88
		2.03	0.689	0.185	41.38
		3.01	0.944	0.250	56.68
	Coiled Fibre	1.00	0.350	0.082	21.00
		2.02	0.670	0.155	40.20
		3.08	0.955	0.215	57.30
Plated Femur	Longitudinal Fibre	1.05	0.311	0.071	18.70
		2.10	0.625	0.118	37.50
		3.02	0.945	0.166	56.70
	Coiled Fibre	1.01	0.358	0.091	21.50
		2.03	0.690	0.183	41.45
		3.11	0.976	0.275	58.60
